# Digital skills among youth: A dataset from a three-wave longitudinal survey in six European countries

**DOI:** 10.1016/j.dib.2024.110396

**Published:** 2024-04-09

**Authors:** Hana Machackova, Marie Jaron Bedrosova, Michal Muzik, Rostislav Zlamal, Jana Fikrlova, Anna Literova, Eliska Dufkova, David Smahel, Hajo Boomgaarden, Hyunjin Song, Petro Tolochko, Leen d'Haenens, Willem Joris, Veronika Kalmus, Mari-Liis Tikerperi, Signe Opermann, Marit Napp, Indrek Soidla, Andre Uibos, Kadri Soo, Katariina Salmela-Aro, Jussi Järvinen, Rasmus Mannerström, Erkki Suvila, Natalia Waechter, Christin Brando, Stepanka Kadera, Giovanna Mascheroni, Davide Cino, Linda Lombi, Alexander van Deursen, Ester van Laar, Jacek Pyżalski, Natalia Walter, Agnieszka Iwanicka, Cristina Ponte, Susana Batista, Rita Baptista, Luc Schneider, Ellen Johanna Helsper

**Affiliations:** a Interdisciplinary Research Team on Internet and Society, Faculty of Social Studies, Masaryk University, Jostova 10, Brno 602 00, Czechia; b Department of Communication, University of Vienna, Kolingasse 14-16, 1070 Vienna, Austria; c Yonsei University, 50 Yonsei-ro Seodaemun-gu, Seoul, 03722, Republic of Korea; d Institute for Media Studies, KU Leuven, Parkstraat 45, 3000 Leuven, Belgium; e Institute of Social Studies, University of Tartu, Lossi 36, 51003 Tartu, Estonia; f University of Helsinki, Yliopistonkatu 3, 00014, Helsinki, Finland; g University of Turku, FI-20014 Turun yliopisto, Finland; h Ludwig-Maximilan University Munich, Department of Educational Science, Leopoldstrasse 13, 80802 Munich, Germany; i Università Cattolica del Sacro Cuore, Largo A. Gemelli, 1, 20123 Milano, Italy; j University of Twente, Drienerlolaan 5, 7522 NB Enschede, the Netherlands; k Adam Mickiewicz University in Poznan, Faculty of Educational Studies, Szamarzewskiego 89, 60-568 Poznan, Poland; l Universidade Nova de Lisboa, Faculty of Social and Human Sciences, *Av*. de Berna, 26 C, 1069-061 Lisboa, Portugal; m Department of Management, London School of Economics and Political Science, Houghton Street, London WC2A 2AE, United Kingdom; n Department of Media and Communications, London School of Economics and Political Science, Fawcett House, 6th Floor, Clements Inn, London, WC2A 2AE, United Kingdom; o ECHO, Vrije Universiteit Brussel, Pleinlaan 9, 1050 Brussel, Belgium

**Keywords:** ySKILLS, Youth, Digital skills, Digital literacy, Longitudinal

## Abstract

This dataset provides longitudinal survey data from a European project, ySKILLS, which was focused on the role of digital skills in youths’ development. It contains data from 10,821 participants from Grades 6–10 (in Wave 1) in Estonia, Finland, Germany, Italy, Poland, and Portugal. The data was collected between Spring 2021 and Spring 2023, the participants were recruited through schools, where the data collection also took place, except for online data collections due to restrictions caused by COVID-19. The dataset is novel in its multidimensional approach to the construct of digital literacy. It provides insight into the development of digital skills in youth and the role of digital skills and internet usage in youths’ positive and negative online experiences and wellbeing. It also contains data that allows for the analysis of the role of digital skills in class networks. The data are beneficial for researchers interested in the examination of youths’ online skills, internet usage, online experiences, and wellbeing from a longitudinal perspective.

Specifications Table

**Table:** 

Subject	Social Sciences
Specific subject area	Digital skills; Digital literacy; Youth; Internet usage; Online experiences; Wellbeing; Class environment;
Data format	SPSS data files format
Type of data	Processed datafile in SPSS data files format
Data collection	Respondents were recruited from schools with a purposive, non-probability sampling method. Data collection occurred over three waves of surveys conducted in 2021, 2022, and 2023, using Computer-Assisted Web Interviews (CAWI). Trained administrators oversaw the survey administration, which took place either in standard computer classrooms or remotely at home during online classes because of the COVID-19 pandemic in 2021. The questionnaire was collaboratively developed by the ySKILLS network and it included a combination of newly devised and validated measures.
Data source location	Institution: KU Leuven City/Town/Region: Leuven Country: Belgium
Data accessibility	Repository name: ySKILLS three-wave survey [1] Data identification number: 10.17632/c66jczxfjc.4 Direct URL to data https://data.mendeley.com/datasets/c66jczxfjc/4

## 1 Value of the Data

**Fig. 1:**
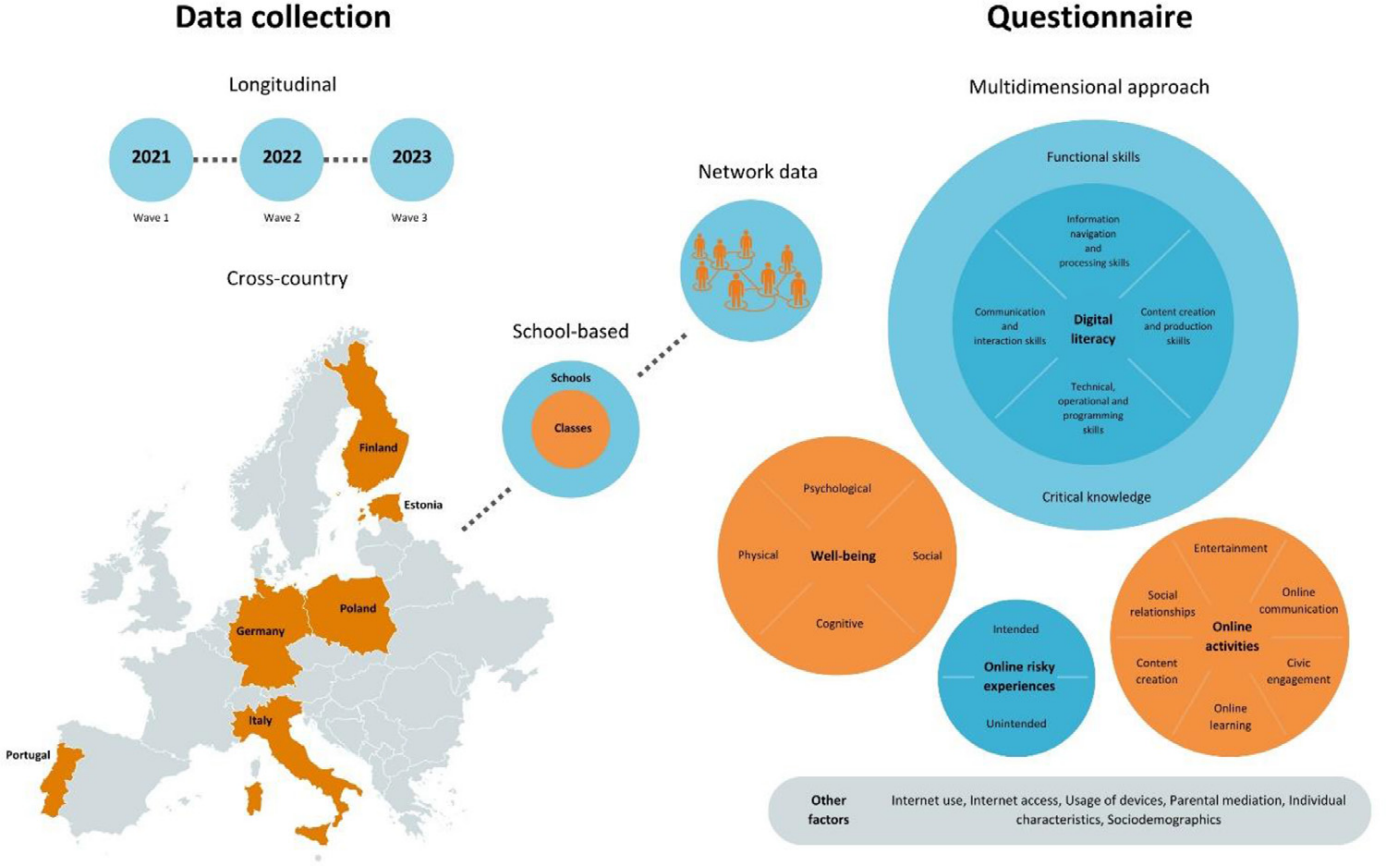
Value of the data.

• The dataset possesses distinctive qualities that make it particularly valuable for the examination of the multidimensional construct of digital skills, combining validated scales related to digital skills and digital knowledge, thereby inferring the overall digital literacy of young people. This comprehensive approach facilitates an in-depth examination of the various dimensions of digital literacy in relation to a multitude of observed online opportunities, online risks, and wellbeing.
• The dataset introduces complexity through its differentiation between intentional and unintentional risky online experiences, encompassing a wide spectrum of online activities and capturing four dimensions of wellbeing (i.e., psychological, social, cognitive, and physical wellbeing).
• The dataset is characterized by its longitudinal panel nature and it is drawn from a robust school-based sample of young people across six European countries that vary geographically and culturally. This longitudinal aspect enables a detailed investigation that goes beyond mere correlational analysis, allowing for comprehensive interpretations of the effects of the factors under scrutiny. The dataset presents unique opportunities for examining the impact of various factors on both the within-person and between-person levels, thereby facilitating a nuanced understanding of the role played by these factors in youths’ development.
• Given the nested structure of the research data, with individuals nested within classes within schools and within countries, it provides the opportunity to investigate the factors across multiple levels. Furthermore, a specific segment of the dataset directly targets the role of digital literacy in relation to effects of class networks. Importantly, this segment of the data also possesses a longitudinal dimension, which contributes novel insights into the network-based data analysis (Fig. 1).

## 2 Data Description

**Fig. 2:**
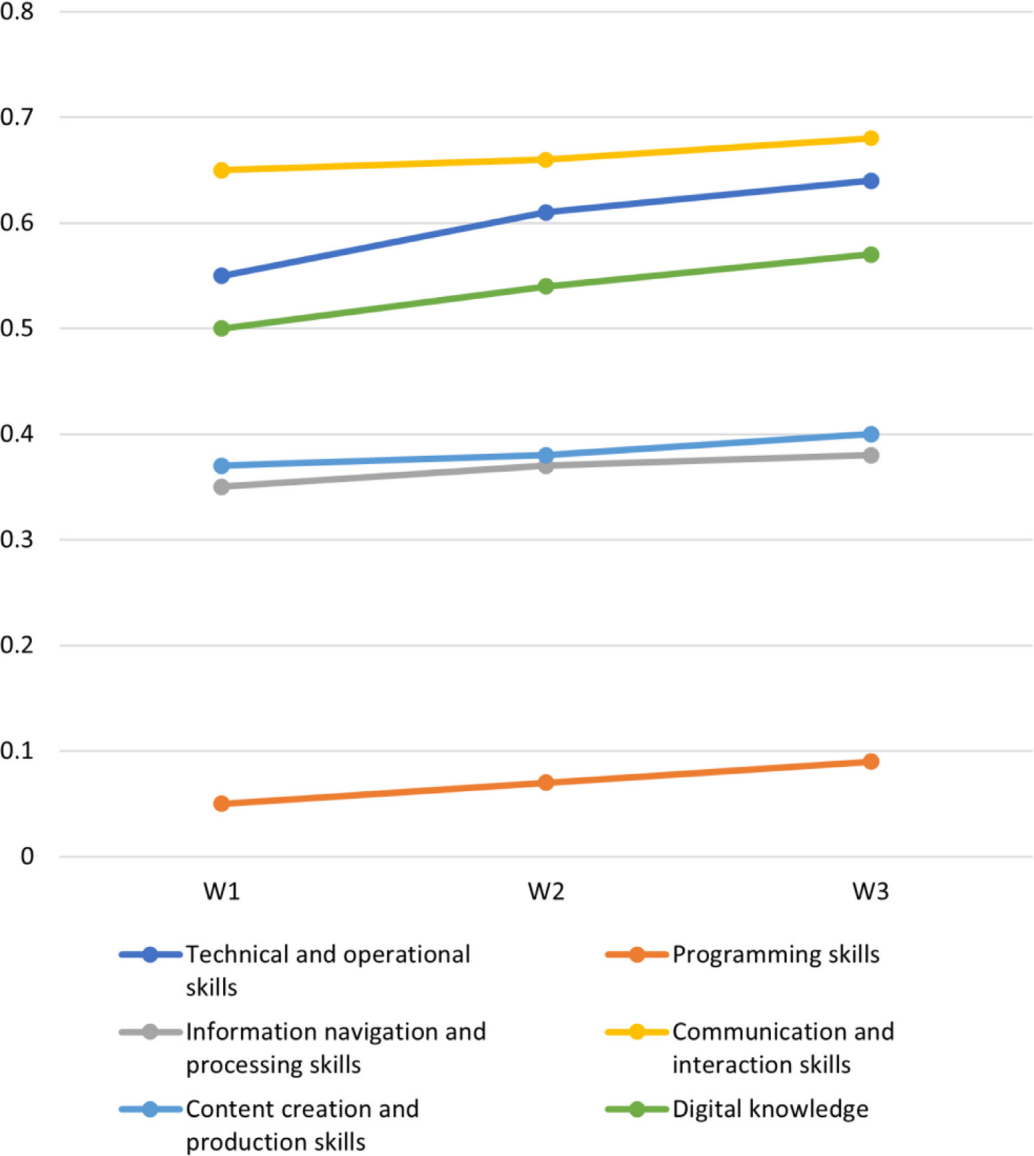
Development of the digital literacy. *Note.* Digital skills are computed as proportions of the particular skill at a high level (i.e. scoring 5 „Very true of me“ on respective items). Digital knowledge is computed as proportion of correct answers in knowledge items.

The dataset contains data derived from a three-wave longitudinal survey conducted across six European countries. The development of the questionnaire was informed by key background information that has been summarized in several publications, including a report that outlines the development and validation of the multidimensional measurement of digital skills [2] (see Fig. 2), a report on the antecedents and consequences of digital skills [3], and a systematic evidence review identifying gaps in our understanding of youths’ digital skills [4]. Based on this background, the questionnaire was crafted to feature a combination of both validated and newly developed measures. To validate the questionnaire, two rounds of cognitive testing were conducted. The initial round took place in August/September 2020, involving 60 participants across all six countries. This phase focused on gauging youths’ understanding of the questionnaire questions, the examples used, and the digital skills items, all of which are integral to the development of this measurement (accessible at [5]). Subsequently, a revised version of the questionnaire was tested in the second round in January/February 2021, with 37 youth participants from the six countries, including 12 participants from the youngest age group who evaluated the questionnaire's length. Expert members of the ySKILLS team in each of the six participating countries coordinated and supervised the translation of the questionnaire. The final versions of the questionnaire, in English and its corresponding national translations, can be accessed online (available at [6]).

The dataset consists of the metadata collected or created by the researchers and self-reported data obtained through the questionnaire. The metadata are detailed in Table 1.

**Table 1:** Metadata variables in the dataset.

Variable name	Description
country	Country of data collection.
waves	Participation in waves.
anonID	Anonymized individual participant's ID code.
startdate	Starting time of data collection.
month_collection	Month of data collection in each wave.
school	Anonymized ID code of participating school.
class	Anonymized ID code of participating class.
school_SES	Socio-economic status of school (varied across countries; not possible to define for Estonia and Finland).
version	Version of the questionnaire.

An overview of self-reported data provided by the participants or variables created from these data is presented in Table 2. These include:

■ Sociodemographic information (age, gender, socioeconomic status, ethnicity).
■ Individual characteristics (perceived discrimination, sensation seeking).
■ Network data (resources, influences).
■ Physical wellbeing (physical health, physical fitness).
■ Psychological wellbeing (life satisfaction, self-efficacy).
■ Social wellbeing (friend support, family support, class environment).
■ Cognitive wellbeing (perceived school performance).
■ Online civic engagement.
■ Parental mediation (restrictive mediation, enabling mediation, monitoring).
■ Internet use (time online, access at home, devices, COVID-19-related access at home).
■ Digital literacy (technical and operational + programming item, information navigation and processing, communication and interaction, content creation and production, distributed over both skills and knowledge items).
■ Online communication (social networking sites use, sharing).
■ Online risks (cyberhate, harmful content, sexting, sexually explicit materials, misinformation and fake news, cyberaggression).
■ Online activities (school and learning, social relationships, entertainment, content creation, internet use for health-related purposes).

**Table 2:** Variables and factors description.

Variable group name	Construct	Variable name	Routing	Description	Values	Derived variables/scales
Sociodemo- graphics	school SES	W#_school_SES_PL - SES_IT		School SES	Categories 1–3	
	age	W#_AGE1a, _AGE1b, _Age_year		Year and month of birth, computed overall age		
	gender	W#_GENDER, _other, _binary		Gender	Categories 0–2; 0–1	
	SES	W#_SES		Financial situation	Categories 1–5	
	ethnicity	W#_ETHN_1_EE – W3_PL_ETHN_other		What language(s) do you speak at home most of the time? Select all, which applies.	A prepared list of local languages including the option “other” (0 – no; 1 – yes)	

Network data	own nickname	W#_NICK		Assigned made-up name (anonymized)	open question	
	standard ego-network name generator questions	W#_NET1a – e_FI		Made-up names for friend 1 - 5 (anonymized)	open question	
	standard ego-network name generator questions	W#_NET2a_rt – 5e_rt_FI	If W#_NET1a – e_FI answered.	Frequency of spending time with friend 1 – 5	Scales 1–7 and 1–5	

Cognitive wellbeing	school performance	W#_COG		School performance during past year	Scale 1–5	

Physical wellbeing	physical health	W#_PHY1		Physical health during past year	Scale 1–5	
	physical fitness	W#_PHY2		Frequency of physical activities during past month	Scale 1–4	

Social wellbeing	friend support	W#_FRIEND1a - c		Measurement of social wellbeing through friend support	Scale 1–4	W#_friends (scale 1–4)
	family support	W#_FAM1a - c		Measurement of social wellbeing through family support	Scale 1–4	W#_family (scale 1–4)
	class environment	W#_CLASS1		Feelings about own class	Scale 1–4	

Psychological wellbeing	life satisfaction	W#_SATI1a - f		Measurement of psychological wellbeing through life satisfaction (positive and negative dimensions)	Scale 1–4	W#_sati_pos (scale 1–4); W#_sati_neg (scale 1–4)

	self-efficacy	W#_EFFI1a - d		Measurement of psychological wellbeing through self-efficacy	Scale 1–4	W#_effi (scale 1–4)

Individual character- istics	perceived discrimination	W#_DISCR		Frequency of experienced own perceived discrimination during past year	Scale 1–6	
	sensation seeking	W#_SENS1a - d		Measurement of sensation seeking	Scale 1–5	W#_sensa (scale 1–5)

Parental mediation	restrictive mediation	W#_MED1a - c		Measurement of restrictive parental mediation of internet use	Scale 1–5	W#_restrict (scale 1–5)
	enabling mediation	W#_MED2a - d		Measurement of enabling parental mediation of internet use	Scale 1–5	W#_enabling (scale 1–5)
	monitoring	W#_MED3a		Parent or carer checks/controls what you do on the internet	Scale 1–5	

Digital literacy	technical and operational	W#_SKILL1a - f		Technical and operation dimension of digital skills	Scale 0–5	W#_skill_tech_pro (scale 0–1)
	programming	W#_SKILL1g		Programming item	Scale 0–5	W#_Skill_progr (categories 0 and 1)
	information navigation and processing	W#_SKILL2a - f		Information navigation and processing dimension of digital skills	Scale 0–5	W#_skill_inf_pro (scale 0–1); W#_lit_inf_pro (scale 0–1)
	communication and interaction	W#_SKILL3a - f		Communications and interaction dimension of digital skills	Scale 0–5	W#_skill_comm_pro (scale 0–1); W#_lit_comm_pro (scale 0–1)
	content creation and production	W#_SKILL4a - f		Content creation and production dimension of digital skills	Scale 0–5	W#_skill_cont_pro (scale 0–1); W#_lit_cont_pro (scale 0–1)
	knowledge items	W#_SKILL5a - f		Knowledge items for digital skills	Categories 0–3	W#_kninf (categories 0–2); W#_kncomm (categories 0–2); W#_kncont (categories 0–2); W#_skill_know_pro (scale 0–1)
	overall skill level	W#_SKILL1a - 4f		Overall skill level (all dimensions of digital skills)	Scale 0–1	W#_skill_overall_pro (scale 0–1)
	overall digital literacy	W#_SKILL1a - 5f		Overall digital literacy level (all dimensions of digital skills ad digital knowledge)	Scale 0–1	W#_litl_know_pro (scale 0–1)

Internet use	time online	W#_INT1		Frequency of spending time online	Scale 1–9	
	devices	W#_INT2a - c		Frequency of internet use on devices	Scale 1–7	
	access at home	W#_INT3		Access to the internet	Categories 0 and 1	
	covid-19	W#_INT4		Inaccessibility of internet during the past	Scale 1–5	

Online activities	online learning	W#_ACT1a - c		Frequency of activities considering online learning during past month	Scale 1–6	W#_daily_activities (scale 0–11)
	social relationships	W#_ACT1d - f		Frequency of activities considering online social relationships during past month	Scale 1–6	W#_daily_activities (scale 0–11)
	entertainment	W#_ACT1g - h		Frequency of online entertaining activities during past month	Scale 1–6	W#_daily_activities (scale 0–11)
	content creation	W#_ACT1i - k		Frequency of activities considering online content creation during past month	Scale 1–6	W#_daily_activities (scale 0–11)

Online communi- cation	SNS use	W#_COM1		Frequency of social network use during past year	Scale 1–7	
	SNS use	W#_COM2_rt	If (2) a few times - (7) almost all the time to item W#_COM1.	Privacy setting on social networks during past year	Categories 0 and 1	
	SNS use	W#_COM3_rt	If (2) a few times - (7) almost all the time to item W#_COM1.	Friend requests from unknown people during past year	Categories 0 and 1	
	sharing	W#_COM4a - c		Sharing information about myself or others on the internet during past year	Scale 1–6	

Civic engagement	online civic engagement	W#_CIV1a - e		Measurement of online civic engagement during past year	Scale 1–4	W#_civic (scale 1–4); W#_civic_dich (categories 0 and 1)

Risks	cyberhate	W#_RISK101 – 106_7_rt2_merged	Items with _rt suffix are routed from previous item	Experience with cyberhate	Categories 0 and 1 or frequencies	
	harmful content	W#_RISK201 – 206_7_rt2_merged	Items with _rt suffix are routed from previous item	Experience with online harmful content	Categories 0 and 1 or frequencies	
	sexting - receiving	W#_RISK301 - 309_11_rt2_merged	Items with _rt suffix are routed from previous item	Experience with sexting - receiving	Categories 0 and 1 or frequencies	
	sexting - sending	W#_RISK312a - b	Items with _rt suffix are routed from previous item	Frequencies of experienced sexting - sending	Scale 1–6	
	sexually explicit materials	W#_RISK401 - 409_11_rt2_merged	Items with _rt suffix are routed from previous item	Experience with online sexual content	Categories 0 and 1 or frequencies	
	fake news - health	W#_RISK501a		Making incorrect decisions about my health, fitness, or dieting	Scale 1–6	
	misinformation and fake news	W#_RISK501b - c		Sharing misinformation	Scale 1–6	
	cybervictimisation	W#_RISK601		Experience with hurtful online treatment	Scale 1–6	
	cyberaggression via sharing	W#_RISK602		Experience with sharing hurtful online content	Scale 1–6	

*Note.* The prefix W# in variable names serves as identifier for the particular wave in which the variables were measured. In the dataset, “W1” is used to indicate variables measured in Wave 1, “W2” for Wave 2, and “W3” for Wave 3.

The dataset incorporates various types of missing values, each denoted by specific codes, including: −99 *Missing value* (indicating a skipped answer); −98 *I do not know*; −97 *I prefer not to sa*y (both options included in the questionnaire); −96 *Routing* (related to online risks, as detailed below); −95 *Cleaning*; and −94 *Not asked*. For a more detailed description of these codes and their use, please refer to the data dictionary provided in the Appendix.

Three distinct categories of factors—network data, online risks, and the digital skills and knowledge indicator—require special considerations in their measurement and computation.

The network data used a system of nicknames. The participants nominated up to three (five in Finland) closest friends (i.e., their nicknames) in their classroom. Then they ranked, on a Likert scale, how often they spend time with each particular friend (1 - “Never” to 7 - “Almost all the time”), how good they think these friends are in using the internet and technologies (1 - “Not good at all” to 5 - “Excellent”), how often they asked that friend for help with using the internet and technologies during this school year (1 - “Never” to 5 - “Daily or almost daily”), and how often these friends asked them for help with using the internet and technologies during this school year (1 - “Never” to 5 - “Daily or almost daily”).

The measurement of online risks was based on a similar measurement from the EU Kids Online project [7], which targeted in more detail experiences with cyberhate, potentially harmful online content, sexting, and sexual content. Each of these risks were measured by several items that were routed subsequently toward each other. Firstly, we asked about having the experience (e.g., cyberhate exposure). Secondly, we asked only those with the experience, how frequently this happened intentionally and unintentionally (the wording „un/expected“ was used for sexting). Thirdly, we asked about the emotional response to the un/intended experience. Therefore, participants without the respective experience (e.g., cyberhate exposure) were not asked the follow-up questions (missing value of −96).

Digital literacy was measured with the youth Digital Skills Indicator (yDSI), which comprises of four subdimensions: technical and operational (including a programming item); information navigation and processing; communication and interaction; and content creation. These subdimensions encompass both functional skills (i.e., the ability to perform tasks) and, with the exception of the technological and operational subdimension, and digital knowledge (i.e., understanding of how online platforms and interactions work). Functional skill items were rated on a Likert scale that ranged from 0 (“I do not understand what this refers to”) to 5 (“very true of me”), while digital knowledge items were evaluated as either “definitely not true” or “definitely true.” Composite scores were generated by computing the proportion of skills a person possesses at a high level (with a value of 5) for skills dimensions and only considering the correct answers for knowledge items.

The dataset includes various types of composites scores, which are used to represent aggregated information, as follows:

- the composite skills indicator measures the proportion of each skill the person has at a high level labelled ‘W#_ skill_[dimension]_pro’
- programming has a dichotomised version of the programming-related item (i.e. only whether the person answered ‘5′, labelled ‘W#_skill_progr’
- the composite knowledge indicator measures the number of knowledge statements that participants answered accurately, labeled ‘W#_kn[dimension]’
- the composite literacy indicator measures the proportion of skills and knowledge at a high level, labelled ‘W#_lit_[dimension]_pro’
- one overall digital skills indicator – proportion of skills at a high level, labelled ‘W#_skill_overall_pro’
- one overall digital knowledge indicator – proportion of knowledge items for which the participants have a correct understanding, labelled ‘W#_skill_know_pro’
- one overall digital literacy indicator – proportion of skills and knowledge at a high level, labelled ‘W#_lit_overall_pro’

For more detailed information regarding the creation and computation of composite measures, please refer to the supplementary materials and syntax for scale creation in the data archive. Additionally, to gain a deeper understanding of the development and validation of the skills and knowledge measures, as well as alternative methods for creating composite measures, please consult the yDSI report [2].

## 3 Experimental Design, Materials and Methods

### 3.1 Sampling

The data collection for this study took place across six European countries: Estonia, Finland, Germany, Italy, Poland, and Portugal. These countries were selected based on their ranking as low, medium, and high on the 2018 Digital Economy and Society Index. A purposive non-probability sampling approach was used to select participants. The target population for this study consisted of adolescents who attended Grades 6 to 10 during Wave 1, which corresponds to secondary schools categorised under ISCED Levels 2 and 3. The aim was to include 1,000 participants per country in Wave 1. To ensure the diversity of the participants, schools were selected based on their socio-economic status, which encompassed varying levels of urbanisation and wealth. To maintain a longitudinal perspective, efforts were made to collect data from the same group of young individuals over multiple waves. This was achieved by surveying the same classes and, if possible, tracking students who transitioned to new schools (this could be done only in some countries). In cases where students departed to new schools and were not reached, new replacement participants were recruited in Wave 2. Typically, this transition occurred when participants were around 14 or 15 years old, corresponding to the transition between ISCED Level 2 and ISCED Level 3.

### 3.2 Data collection

The data collection process unfolded in three waves during the spring of 2021, 2022, and 2023. However, in specific cases, such as in Finland, Italy, and Poland during Wave 1, the data collection had to be adjusted due to the impact of COVID-19 pandemic restrictions. In these countries, data collection in several schools was postponed and conducted in the autumn of 2021 (see [8]). The methodology employed for data collection involved Computer-Assisted Web Interviewing (CAWI), which took place in computer classrooms with trained administrators overseeing the process. In instances where schools were closed due to the COVID-19 pandemic, data collection was conducted through online classes, either from the participants’ homes or through a hybrid approach, again under the supervision of trained administrators. The data collection sessions were designed to fit within the standard school period and typically lasted less than 45 min. This approach ensured minimal disruption to the regular school routine.

### 3.3 Sample description

The sample description by wave and country is displayed in Table 3. The distribution of age and gender is presented in Table 4. To maintain continuity across all three waves, participants were connected through their unique identification codes, which were either self-generated or assigned by the researchers. However, not all data could be successfully linked across all waves due to several factors, including the recruitment of new participants in Wave 2, instances of schools or classes dropping out of the study, and errors made by participants in recording their identification codes. The information about data across all three waves are detailed in Table 3.

**Table 3:** Sample size by wave and country (N).

Participation in waves	Only W1	Only W2	Only W3	W1 and W2	W1 and W3	W2 and W3	W1 and W2 and W3	Total
Estonia	312	191	207	228	104	211	606	1,859
Finland	99	91	687	186	62	176	441	1,742
Germany	365	221	281	207	108	192	403	1,777
Italy	265	360	201	329	22	743	351	2,271
Poland	469	433	169	346	81	176	261	1,935
Portugal	242	44	11	180	28	134	598	1,237
Total	1,752	1,340	1,556	1,476	405	1,632	2,660	10,821

*Note.* W1 = Wave 1. W2 = Wave 2. W3 = Wave 3**.**

**Table 4:** Age and gender distribution by wave and country.

	Wave 1					Wave 2					Wave 3				
	Age		Gender			Age		Gender			Age		Gender		
	*M*	*SD*	% girls	% boys	% other	*M*	*SD*	% girls	% boys	% other	*M*	*SD*	% girls	% boys	% other
Estonia	14.65	1.24	48.72	49.52	1.76	15.52	1.20	49.19	47.57	3.24	16.45	1.17	46.37	50.35	3.28
Finland	14.44	0.97	46.16	51.28	2.56	15.41	1.14	46.15	50.34	3.51	16.07	1.01	45.79	50.34	3.88
Germany	14.08	1.33	52.63	46.35	1.02	14.97	1.21	51.91	46.14	1.96	15.85	1.14	51.88	45.57	2.54
Italy	14.39	1.18	57.50	41.16	1.34	15.67	1.07	50.25	47.39	2.36	16.82	0.94	45.86	51.40	2.73
Poland	14.08	1.44	46.71	49.65	3.63	14.96	1.50	49.26	45.39	5.35	15.88	1.78	43.67	48.76	7.57
Portugal	14.65	1.29	49.71	50.29	0.00	15.59	1.24	49.42	50.16	0.42	16.55	1.22	49.03	50.32	0.65
Total	14.38	1.29	50.22	48.06	1.72	15.38	1.26	49.51	47.64	2.85	16.30	1.23	47.04	49.64	3.32

The attrition rates observed in this study from Wave 1 to Wave 2 amounted to 34.3%. From Wave 2 to Wave 3, the attrition rate increased to 39.6%. Consequently, the cumulative attrition from Wave 1 to Wave 3 was 54.8%. To evaluate the impact of attrition on the sample, we conducted tests to assess the differences in key variables, including age, gender, SES, internet use, and subdimensions of the digital skills indicator, across the waves. Importantly, these tests revealed that the differences in the sample due to attrition were minimal. Specifically, for gender, the Cramer's *V* values ranged between 0.029 and 0.034, indicating negligible differences (threshold for small effect *V* = 0.1). Similarly, for other variables, such as age, SES, internet use, and the subdimensions of the digital literacy scale, the Cohen's *d* values ranged from −0.153 to 0.085, underscoring that any observed variations were of a small magnitude (threshold for small effect *d* = 0.2).

### 3.4 Versions of the questionnaire

The questionnaire was distributed in four distinct versions, each varying in terms of the inclusion of risk-related questions and a dedicated network section. The versions varied for younger (approximately Grades 6–8) and older (approximately Grades 9–10) age groups. This decision was driven by both the length of the questionnaire and the sensitive nature of certain items. For reference, a detailed breakdown of the distributions of these questionnaire versions can be found in Table 5.

**Table 5:** Questionnaire versions by country and age group.

Country	Version	Targeted age group in W1	Network data section	Risk section
Estonia	B	younger and older	no	yes

Finland	A	younger and older	yes	yes

Germany	A	older	yes	yes
	C	younger	yes	no

Italy	A B	older younger	yes no	yes yes

Poland	B	younger and older	no	yes

Portugal	A; B	older	yes (A); no (B)	yes
	C; D	younger	yes (C); no (D)	no

## Limitations

The process of data collection faced challenges posed by the COVID-19 pandemic and associated lockdown measures. Consequently, data collection for the first wave (W1) had to be adapted to the circumstances, necessitating the collection of data from participants in their homes (see [8]).

## Ethics Statement

The authors adhered to the ethical requirements for publication in *Data in Brief* and confirm that the research did not involve any animal experiments and that data collected from social media platforms was not a component of this study. Moreover, the research was conducted in strict accordance with the principles outlined in the Declaration of Helsinki, which emphasizes the ethical conduct of research involving human subjects. Ethical approval for the study was obtained by the IBR committee of the project coordinator's university (KU Leuven) (Application Dossier Social and Societal Ethics Committee, 2020). The project partners responsible for the data collection in their countries applied for ethical approval according to national regulations (for further details and country specifics, see [8]: 309). To ensure that ethical standards were maintained, informed consent was sought from participants (and their parents or legal representatives), either through active or passive means, prior to their involvement in the study, thus demonstrating a commitment to upholding ethical considerations throughout the research process.

## CRediT authorship contribution statement

**Hana Machackova:** Conceptualization, Methodology, Data curation, Writing – original draft, Supervision, Project administration. **Marie Jaron Bedrosova:** Conceptualization, Methodology, Data curation, Writing – original draft, Supervision. **Michal Muzik:** Formal analysis, Data curation, Writing – original draft. **Rostislav Zlamal:** Data curation. **Jana Fikrlova:** Data curation. **Anna Literova:** Data curation. **Eliska Dufkova:** Data curation. **David Smahel:** Conceptualization. **Hajo Boomgaarden:** Conceptualization, Writing – review & editing. **Hyunjin Song:** Conceptualization. **Petro Tolochko:** Conceptualization. **Leen d'Haenens:** Funding acquisition, Project administration, Supervision, Writing – review & editing. **Willem Joris:** Funding acquisition, Project administration, Supervision. **Veronika Kalmus:** Conceptualization, Investigation, Writing – review & editing, Supervision, Project administration. **Mari-Liis Tikerperi:** Investigation, Project administration. **Signe Opermann:** Investigation. **Marit Napp:** Investigation. **Indrek Soidla:** Investigation, Data curation. **Andre Uibos:** Investigation. **Kadri Soo:** Investigation. **Katariina Salmela-Aro:** Supervision, Conceptualization. **Jussi Järvinen:** Project administration, Data curation. **Rasmus Mannerström:** Project administration, Conceptualization, Data curation. **Erkki Suvila:** Data curation. **Natalia Waechter:** Methodology, Conceptualization, Investigation, Data curation, Writing – review & editing, Supervision, Project administration. **Christin Brando:** Methodology, Investigation. **Stepanka Kadera:** Investigation. **Giovanna Mascheroni:** Conceptualization, Investigation, Supervision, Project administration. **Davide Cino:** Investigation. **Linda Lombi:** Investigation. **Alexander van Deursen:** Conceptualization, Formal analysis, Investigation, Methodology, Supervision, Validation. **Ester van Laar:** Conceptualization, Formal analysis, Validation. **Jacek Pyżalski:** Supervision, Project administration, Investigation, Data curation, Writing – review & editing. **Natalia Walter:** Investigation, Data curation. **Agnieszka Iwanicka:** Investigation, Data curation. **Cristina Ponte:** Project administration, Investigation. **Susana Batista:** Supervision, Investigation. **Rita Baptista:** Investigation. **Luc Schneider:** Conceptualization, Formal analysis, Validation. **Ellen Johanna Helsper:** Conceptualization, Formal analysis, Investigation, Methodology, Supervision, Validation, Writing – original draft, Writing – review & editing.

## Data Availability

ySKILLS three-wave survey (Original data) (Mendeley Data)
